# Outcome of Partial Adrenalectomy in MEN2 Syndrome: Personal Experience and Systematic Review of Literature

**DOI:** 10.3390/life13020425

**Published:** 2023-02-02

**Authors:** Priscilla Francesca Procopio, Francesco Pennestrì, Carmela De Crea, Nikolaos Voloudakis, Rocco Bellantone, Marco Raffaelli

**Affiliations:** 1 Centro di Ricerca in Chirurgia delle Ghiandole Endocrine e dell’Obesità, Università Cattolica del Sacro Cuore, 00168 Roma, Italy; 2 U.O.C. Chirurgia Endocrina e Metabolica, Centro Dipartimentale di Chirurgia Endocrina e dell’Obesità, Fondazione Policlinico Universitario Agostino Gemelli IRCCS, 00168 Roma, Italy; 3 U.O.C. Chirurgia Endocrina, Fatebenefratelli Isola Tiberina-Gemelli Isola, 00186 Roma, Italy

**Keywords:** pheochromocytoma, MEN2, clinical outcome, recurrence, corticosteroid therapy, partial adrenalectomy

## Abstract

*Background*: Partial adrenalectomy (PA) is an alternative option to total adrenalectomy for the treatment of hereditary pheochromocytoma (PHEO) to preserve cortical function and avoid life-long steroid replacement. The aim of this review is to summarize current evidence in terms of clinical outcome, recurrence, and corticosteroid therapy implementation after PA for MEN2-PHEOs. *Material and Methods*: From a total of 931 adrenalectomies (1997–2022), 16 of the 194 patients who underwent surgical treatment of PHEO had MEN2 syndrome. There were six patients scheduled for PA. MEDLINE^®^, EMBASE^®^, Web of Science, and Cochrane Library were searched for English studies from 1981 to 2022. *Results*: Among six patients who underwent PA for MEN2-related PHEO in our center, we reported two with bilateral synchronous disease and three with metachronous PHEOs. One recurrence was registered. Less than 20 mg/day Hydrocortison therapy was necessary in 50% of patients after bilateral procedures. Systematic review identified 83 PA for MEN2-PHEO. Bilateral synchronous PHEO, metachronous PHEO and disease recurrence were reported in 42%, 26%, and 4% of patients, respectively. Postoperative steroid implementation was necessary in 65% of patients who underwent bilateral procedures. *Conclusions*: PA seems to be a safe and valuable option for the treatment of MEN2-related PHEOs, balancing the risk of disease recurrence with the need for corticosteroid therapy.

## 1. Introduction

Pheochromocytoma (PHEO) is a neuroendocrine tumor arising from adreno-medullary chromaffin cells, which can display diverse phenotypes, and the hereditary forms can occur in 20–30% of cases [1,2,3]. The peak incidence of the disease occurs during the fourth and fifth decades of life, though it presents earlier in case of hereditary syndromes, with the same ratio of males to females [4].

These tumors may lead to life-threatening conditions when untreated [4,5]. As PHEOs manifest with bilateral clinical presentation in up to 72% of hereditary and 25% of non-hereditary cases, complete surgical excision, together with systemic blood pressure medications, has been traditionally considered the definitive treatment of the disease [6,7,8,9,10]. Indeed, as a consequence of the (synchronous or metachronous) bilaterality of the disease in the hereditary forms, bilateral total adrenalectomy (TA) is still used in many centers, either as a one step or two-step procedure [11,12]. This may lead to the risk of adrenal insufficiency and demand for life-long steroid replacement, which is associated with a decreased quality of life [7].

In order to avoid, or at least reduce, the need for life-long corticosteroid therapy, partial adrenalectomy (PA) has been proposed as a valuable alternative to TA. This approach is based on three assumptions: infrequency of malignant PHEO in some inherited syndromes, acceptable risk of recurrence (7% over 3 years and 10–15% over 10 years) and high likelihood of corticosteroid independence [8,13]. Risk of recurrence in such cases seems to be of lesser significance when compared to the morbidity and mortality of adrenal insufficiency, consequent lifelong hormone replacement and its deleterious effect on puberty and quality of life [9]. Indeed, hereditary PHEOs have an earlier onset and are not uncommon in children and adolescents. Thus, maintaining the adrenocortical function is essential for the normal development of young patients [8]. Malignancy features of the disease also validate the choice of partial procedures. Indeed, while unilateral TA is generally recommended in the sporadic setting, where the presumed malignancy risk is about 10%, in most of hereditary PHEO malignancy rate is low, with one international study identifying only two malignancies among 563 patients with multiple endocrine neoplasia type 2 (MEN2) [14].

Therefore, the selection of patients who will benefit the most from PA, as a component of bilateral surgical treatment, is critical for ensuring good outcomes [15]. In MEN2-related PHEOs, the specific type of RET mutation is relevant for the planning of surgical strategy: in families with a low incidence of malignancy PA surgery may represent a safe and feasible option to avoid steroid dependency [7,9,10,13,16]. In these patients the importance of preserving normal adrenal tissue is underscored by the risk of decreased quality of life associated with chronic exogenous steroid replacement [17].

Given the above considerations, PA strategies have been more recently accepted as a valuable alternative for the treatment of bilateral disease and in patients with PHEOs related to genetic syndromes, including MEN2 [7,9,10,13]. However, PA should still be considered as a technically demanding surgical procedure, which may expose the patients to the risk of recurrence [18]. The evaluation of the amount of residual adrenal cortical tissue to be preserved during surgery is a crucial factor to maintain an acceptable cortical function while ensuring an adequate decrease in hormonal hypersecretion and tumor clearance [19,20]. Therefore, the surgical management of MEN2-related PHEOs should be tailored to patients’ phenotype features and balance the risk of disease recurrence with the need of steroid treatment [16,21].

The aim of this review is to summarize current evidence related to MEN2 patients who underwent PA, in terms of clinical outcome, recurrence and need for corticosteroid therapy, and compare our experience with the published literature.

## 2. Material and Methods

### 2.1. Our Experience

#### 2.1.1. Study Population

We retrospectively reviewed all patients requiring adrenalectomy from January 1997 to October 2022 in our center and collected data from those who underwent partial procedures for MEN2-related PHEO in a specifically designed database.

Hospital records and operative reports were reviewed for demographic and perioperative data. Baseline patients’ characteristics included gender, age, genetic mutation and previous adrenal surgeries. Preoperative characteristics included hormonal status, as well as tumor side and size. The preoperative workup included clinical, biochemical, radiological and nuclear evaluation according to international society guidelines [22]. Genetic tests were also performed according to the specific clinical scenario. Operative parameters consisted of surgical approach, operative time, intraoperative complications and conversion rate. Postoperative pathological evaluation confirmed the diagnosis. Postoperative parameters included histopathology, length of hospital stay, early complications, readmission, disease recurrence, mortality and corticosteroid therapy.

All operations were performed after at least 2 weeks of biochemical blockade using doxazosine to guarantee an appropriate blood pressure control, according to the main clinical guidelines [22]. Beta blockers were also administrated pre-operatively, in selected cases, to control tachycardia. Postoperative corticosteroid supplementation was administrated in patients who underwent bilateral adrenalectomy (as a single step procedure or the second step after unilateral adrenalectomy).

All partial procedures were performed by an expert endocrine surgeon (M.R.).

Follow-up consisted of either out-patient appointment or telephone consultation. For this study, follow-up evaluation ended on 30 November 2022.

No approval by the ethical committee of our institution was necessary, as data for this retrospective study have been institutionally collected from patients who had signed the consent for rare endocrine neoplasm at the time of hospitalization (Eurocrine^®^ registry for rare endocrine).

#### 2.1.2. Definitions

According to the World Health Organization (WHO) classification of endocrine tumors of 2014, *PHEO* is defined as an intra-adrenal paraganglioma (PPG), whereas the term extra-axial PPG is applied for PPGs with an extra-adrenal location [9]. However, the WHO’s conceptualization of PHEO and PPG has evolved in parallel with changing concepts of the development and function of normal paraganglia and with concomitant changes in terminology [23]. Indeed, the arbitrary separation of these groups of tumors with the concomitant acknowledgement of their similarities is clear [23]. In recognition of such a paradox, a subtle terminology change is seen in the 2022 WHO bluebooks, so PHEO is now defined as an intra-adrenal PPG that originates from chromaffin cells of the adrenal medulla [23].

*Hereditary PHEO* can occur in 20–30% of patients [1,2]. Indeed, PHEO can develop in association with several familial neoplastic syndromes, including von Hippel Lindau (VHL) syndrome, neurofibromatosis type 1 (NF1), and MEN2 [6]. MEN2A and MEN2B represent 95% and 5% of all MEN2, respectively, with a similar penetrance of PHEO in both syndromes (about 50% of cases) [16,21,24]. Moreover, bilateral clinical presentation may occur in 3%–25% of all patients and in 50–60% of MEN2-patients [2,3,17,21,25,26,27,28]. Bilateral MEN2-PHEOs development is more frequently metachronous rather than synchronous. More specifically, metachronous PHEOs have been reported in up to 25% of cases after a mean period of 5–15 years, though the delay between the first and second PHEO can be highly variable [21,26,27,28].

Until 2022 *malignancy* used to be defined by the presence of metastases in nonchromaffin tissue, which were generally found in patients with large tumors [8,22,26]. However, according to the more recent classifications, all PHEOs (and PGLs) should be considered as malignant neoplasms with variable metastatic potential similar to epithelial neuroendocrine tumors [23]. Indeed, while these tumors could frequently be detected and surgically treated at an early stage due to symptoms resulting from catecholamine overproduction, the clinical course could not be predicted [23]. This uncertainty has led to long periods of follow-up, and metastases have been reported even 20 years following initial surgery [23]. Several attempts have been made to develop scoring or classification systems for predicting the risk of metastasis [23]. The 2022 WHO classification of PGLs does not endorse any of these systems, but at the same time, it does not discourage their use in individual practices [23].

*Minimally-invasive adrenalectomy* through the laparoscopic transabdominal lateral approach (TLA) was introduced in our center in 1997. Subsequently, posterior retroperitoneoscopic approach (PRA) was introduced in 2003, followed by robotic surgery through a transabdominal lateral approach in 2012. Robotic procedures have been performed by means of both robotic platforms: Da Vinci (Da Vinci system, Intuitive Surgical, Sunnyvale, CA, USA) (since 2012) and Hugo™ RAS (Medtronic, Minneapolis, MN, USA) (since 2022). Surgical technique has already been described in previous publications in detail [29,30,31,32,33].

*PA* is defined as the removal of the part of the adrenal gland, including the disease, while leaving a portion of vascularized tissue behind. Vascularization may be evaluated on the basis of the surgeon’s judgement or by means of intraoperative adjuncts, such as Indocyanine Green (ICG). Unilateral PA is defined as the surgical excision of a portion of one adrenal gland. Bilateral procedures include bilateral PAs (defined as the removal of one portion of both adrenals) and unilateral PA with contralateral TA.

The *operative time* is defined as the interval from incision to wound closure (skin to skin).

The severity of *postoperative complications* was graded according to the Clavien–Dindo classification.

*Intraoperative complications* were defined as all the events that could potentially cause injury and require unplanned surgical maneuvers.

*Postoperative complications* were defined as any event altering the regular postoperative course, and/or delaying discharge, occurring until the 30th postoperative day (POD).

*Mortality* was defined as any intraoperative or postoperative death within 30 days of surgery.

*Follow-up time* was defined as the time interval between the date of the surgical procedure and the date of the last follow-up examination, consisting of radiological and/or nuclear medical evaluation, clinic visits, plasma and 24-h urinary catecholamine and metanephrine levels.

### 2.2. Systematic Review

#### 2.2.1. Search Strategy

The review process was conducted according to the Preferred Reporting Items for Systematic Reviews and Meta-Analyses (PRISMA) guidelines [34]. No ethical approval was necessary, as only data from previous published studies were analyzed. Methods of analysis and inclusion criteria were defined in advance and outlined in the designated protocol.

The main terms used to perform the data search were pheochromocytoma, MEN2, clinical outcome, recurrence, corticosteroid therapy, partial adrenalectomy (as well as subtotal, adrenal-/organ-/cortical-preserving, and adrenal-/organ-/cortical-sparing adrenalectomy, to cover the variability of reporting terms in the literature).

Comprehensive systematic searches in recognized electronic, bibliographic medical databases, such as MEDLINE^®^ (Pubmed), EMBASE^®^, Web of Science and Cochrane were performed. References were examined among articles published in English from 1 January 1981 till 1 January 2022.

#### 2.2.2. Eligibility Criteria

Eligibility criteria for the systematic review were based on the following inclusion criteria: patients of all ages with diagnosed pheochromocytoma and MEN2 syndrome, regardless of the presence of metastatic disease, who underwent unilateral or bilateral PA; articles dealing with clinical outcomes after PA in terms of disease recurrence and corticosteroid therapy. RCTs, quasi-RCTs, cross-sectional studies, retrospective and prospective cohort studies, case-control studies, and case series were included.

The exclusion criteria for the systematic review were the following: animal studies, languages different from English, case reports, conference or congress abstracts, comments/editorial letters, guidelines, reviews and meta-analyses.

With the aim of providing a more detailed report of the topic in the descriptive sections of the review, the eligibility criteria were expanded to include: guidelines, reviews and meta-analyses; articles dealing with the correlation between the genetic mutations and the incidence of PHEO, the severity of the disease, and the risk of recurrence; articles dealing with clinical outcomes after TA, in terms of recurrence and corticosteroid therapy.

#### 2.2.3. Study Selection and Quality Assessment

**Figure 1:**
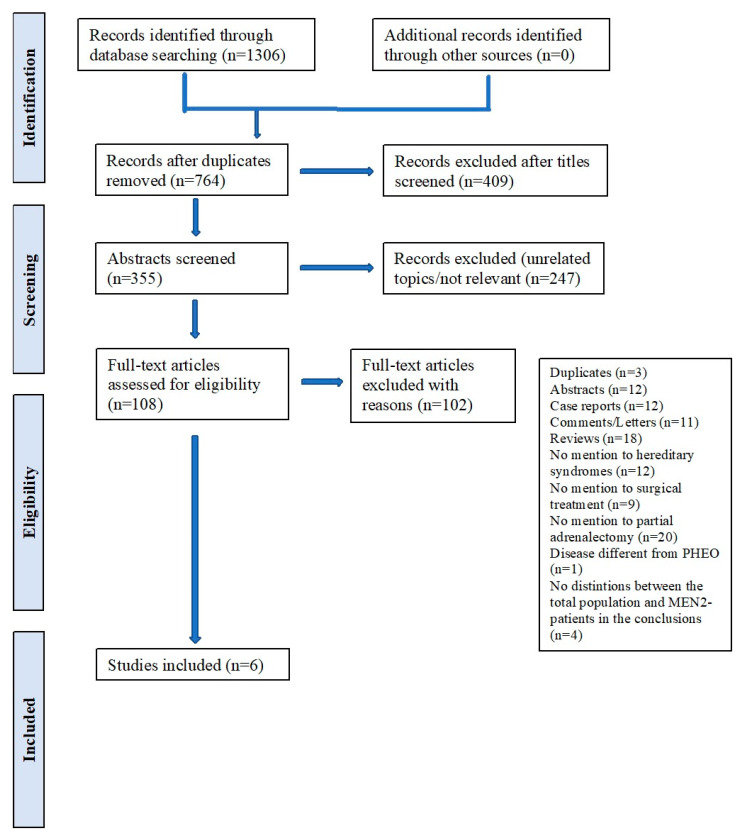
PRISMA flowchart.

Our literature search generated a total of 1306 articles, of which 764 duplicates were discarded. Abstracts were independently selected by two reviewers (P.F.P. and F.P.), and discrepancies were resolved via discussion with a third reviewer (M.R.). Subsequently, 108 full-text articles were considered, and those meeting the inclusion criteria were re-evaluated for eligibility. Finally, six articles were selected for our review and relevant data were collected in a specifically constructed Microsoft Excel file (P.F.P. and F.P.). The studies selection process has been summarized in a PRISMA flow-chart (Figure 1).

## 3. Results-Comparison with Our Experience

From a total of 931 adrenalectomies (including 46 bilateral procedures), 194 patients required surgical treatment of PHEO (20%) (65 laparotomic approach, 68 TLA, 35 PRA, 25 RA).

Out of 194 patients (8.2%), 16 who underwent adrenalectomy for PHEO suffered from MEN2 syndrome (10 MEN2A, 6 MEN2B). Out of 16 patients (37%), 6 underwent PA: demographic characteristics, lesion site, surgical procedure, post-operative and clinical outcome are summarized in Table 1. Mean age at surgery was 36 ± 8.9. There was one patient treated with PA for unilateral PHEO. There were two patients presented with bilateral synchronous disease at the time of referral and underwent unilateral TA and contralateral PA. There were three patients who developed metachronous disease, after a median of 84 months from the primary surgical procedure (unilateral TA), requiring a contralateral PA. Among these patients, one developed recurrence (on the right side) 48 months after the previous partial procedure, requiring complete adrenalectomy. No further disease recurrences were reported. Evaluation of the remnant vascularization was performed by means of intraoperative ICG angiography in four cases (two bilateral procedures and two unilateral procedures), showing the viability of the left adrenal tissue. After a mean 19.6 ± 21.6 months follow-up, all patients who underwent bilateral (one or two-step) procedures required Hydrocortison therapy (mean dosage 20 ± 10 mg/day). No Addisonian crises were registered during the follow-up.

We compared our results with the outcome of our systematic review (Table 2). From a total of 218 patients who underwent PA for PHEO, we analyzed the clinical presentation of the disease and surgical treatment of 83 (38%) patients with MEN2 syndrome. Median age at surgery was 35. Disease recurrence was reported in four patients (4%). There were 35 patients (42%) who had bilateral synchronous PHEOs at the time of referral, while 22 patients (26%) required further surgical treatment for metachronous disease.

In order to evaluate the clinical outcome, in terms of post-surgical need of steroid implementation, we analyzed the bilateral adrenalectomies (both in synchronous and metachronous PHEOs) (Table 3). Among a total of 58 adrenalectomies, 35 procedures (60%) were performed due to bilateral synchronous disease at the time of referral (8 unilateral TAs and contralateral PAs, 22 bilateral TAs, 5 bilateral PAs). Recurrence was reported in 3 cases (5%), while 22 patients (37%) presented metachronous disease. Surgical treatment was achieved by means of contralateral PA, contralateral TA, and bilateral TA in 8, 14, and 1 cases, respectively. At a medium follow-up of 36 months, steroid implementation was necessary in 38 patients (65%).

We compared our center’s results with the aforementioned data from the literature: two patients were treated with unilateral TA and contralateral PA for synchronous PHEOs. There were three contralateral PAs performed due to metachronous PHEOs. Among these patients, one also underwent ipsilateral TA for disease recurrence. All patients who underwent bilateral procedures required post-surgical steroid therapy.

## 4. Discussion

PA is a safe and effective alternative to TA in hereditary forms of PHEO [20]. Several different terms have been used for this approach, (partial, subtotal, adrenal-/organ-/cortical-preserving, and adrenal-/organ-/cortical-sparing adrenalectomy) [37]. Macroscopic intraoperative distinction between the cortex and the medulla is a myth, even with endoscopic magnification [37]. Consequently, the term “cortical-sparing” is not literally correct and should be avoided [37].

Since the first published experiences, minimally-invasive PA has been proposed in case of bilateral adrenal tumors to preserve cortical function and avoid life-long steroid replacement [20]. Indeed, acute adrenocortical insufficiency after BA occurs in up to 23% of patients, even with corticosteroid therapy, while concurrent risks of corticoid overreplacement (such as diabetes, hypertension, osteoporosis) remain, signifying that reaching a dosage equilibrium may prove challenging in clinical practice [38].

PA was first described in 1934 by De Courcy and then introduced in 1983 to treat familial bilateral PHEO by Irvin et al. [10,20,39] Minimally-invasive approaches (PRA) to PA in PHEO patients were first reported by Walz et al. [20] (1996), achieving good results in terms of surgical outcome and residual cortical function. In 2004, the same team also reported the largest series in the published literature with 100 laparoscopic PA procedures [36,40]. Following that, Sasagawa et al. [41] also concluded that PA via PRA constitutes a safe and less invasive method for the treatment of adrenal tumors, including PHEOs.

A literature review by Kaye et al. [42] observed that the use of PA is increasing worldwide and does not carry additional morbidity compared to TA. Less blood loss and shorter operative times during adrenal partial procedures have been reported when compared with whole-organ removal [40,42]. Alesina et al. [13] suggested that PA should always be contemplated in case of PHEO, especially when considering that familial diseases account, nowadays, for almost 40% of all cases. Kawasaki et al. [43] applied PA surgery for MEN2A PHEOs, whereas for MEN2B, bilateral TA was performed based on their experience of stronger association with multicentricity and aggressive behavior in such cases.

To date, TLA is the most used surgical approach worldwide; however, it requires patient repositioning in bilateral procedures [20]. PRA may represent a valid option to avoid this disadvantage, taking into consideration that patient repositioning, apart from being time consuming, can also cause excessive stress in PHEO patients, leading to anesthesiologic adverse events, such as hypertensive crisis [20]. Moreover, by exposing both adrenal lodges simultaneously, the prone position presents technical advantages in BA cases [31].

Endorsement of robotic technologies and fluorescence techniques in adrenal surgery, may further improve PA results and consequently avoid unnecessary TA [44]. Indeed, one of the advantages of the robotic approach in PA consists of reducing gland manipulation, thus preserving the vascularization of the residual adrenal tissue and overcoming some limitations of the conventional laparoscopic approach [44].

Although the feasibility and safety of the laparoscopic approach to BA has been well-documented, lifelong steroid replacement remains a problem when both adrenal glands are totally removed, whether performed synchronously or metachronously [45]. The commonly encountered adverse side effects with chronic steroid dependence are well-defined [11,12,38]. Indeed, when acute hypocortisolism is not promptly corrected with hospital admission and administration of intravenous saline and corticosteroids, life-threatening clinical manifestations may occur [12,43]. On the other hand, excessive steroid replacement is associated with premature osteoporosis, hypertension, and diabetes [11].

When bilateral adrenal tumors are encountered, unilateral or bilateral PA seems to be a preferable choice if part of functioning adrenal tissue can be preserved [21,45]. Although laparoscopic simultaneous bilateral PA might increase the operative duration and technical complexity over unilateral procedures, it has been reported as a successful procedure in patients with bilateral PHEOs [45]. Tumor size is a relevant factor for selecting bilateral PAs for bilateral functioning tumors [45]. In contrast with unilateral PAs, bilateral procedures seem to have more advantages if discrete tumor masses can be localized and identified in each adrenal gland [45]. On the other hand, PA results are less favorable in large tumors with little normal adrenal tissue [45]. Ihara et al. [45] suggested PA surgery for bilateral adrenal tumors in case of lesion dimension ≤3 cm in diameter on either side. Liao et al. [45] also reported a series of patients who underwent bilateral PA: the lesion size range was 1.0–2.5 cm, and more than a third of the normal adrenal tissue could be preserved. Similar conclusions were reached by Scholten et al. [12], suggesting that large tumors with unfavorable locations may preclude sparing of a significant amount of the adrenal cortex.

The extent of the surgical resection is determined not only by tumor size but also location in relation to vascular supply [12]. Indeed, to date, the main technical concerns about this particular kind of surgical procedure are the management of the main adrenal vein and the resection margins [20]. The main adrenal vein division is considered mandatory in case of PHEO, since the vein directly originates from the adrenal medulla [20]. On the other hand, its division may compromise venous drainage from the adrenal stump and, consequently, impair cortical remnant function [20]. Despite such concerns, the adrenal vein preservation-related risk of postoperative steroid dependence is still unclear [2]. Several authors report acceptable cortical function even in patients submitted to bilateral PA with bilateral division of the adrenal vein [2]. It seems that, when the remnant of the adrenal gland is left in situ, without excessive and unnecessary mobilization, there are sufficient collateral arteries and veins providing vascular supply [20]. Use of ICG has been described as a useful technique to subjectively note the perfusion of the remnant adrenal gland following resection [2,44]. Exhibition of fluorescence is dependent on the histological tissue features (medullary vs. cortical). Moore et al. [46] reported that, on multivariate analysis, origin of adrenal cortical tissue was the only predictor of hyperfluorescence following ICG administration (95%, 33%, and 50% for tumors of adrenocortical, medullary and other tissue origins, respectively). Especially in medullary lesions, which are hypofluorescent in contrast to the surrounding adrenal tissue, ICG may delineate tumor margins and enable a more precise excision and, subsequently, increase the amount of remnant adrenal tissue [46].

The amount of residual adrenal cortical tissue to spare during surgery, in order to maintain an acceptable cortical function while ensuring adequate tumor clearance, has been debated [19,20]. As shown by Brauckhoff et al. [15], avoiding corticosteroid dependence may be achieved by preserving only a small portion of the total functional adrenal cortex. Current data estimate that 15%–30% of adrenal tissue must remain in situ for adequate function, although there is no definitive data regarding the amount of tissue needed to preserve and maintain physiologic adrenal function [17,19].

A small remnant leads to the double risk of recurrence and adrenal insufficiency [8]. It has been shown that the remnant functional recovery is time-dependent, and its volume is correlated with stress capacity [8]. Although the minimum adrenal volume for intact adrenocortical function cannot be defined, a longer functional recovery may be expected in patients with smaller remnants [8]. Nagaraja et al. [19] reported, in a meta-analysis, that the overall steroid-independent rate of patients who underwent PA was 85%, while Liao et al. [19,45] stated that, after bilateral PA, life-long steroid supplements were not needed in all patients during the 61-month follow-up. In a recent review of 179 patients who underwent PA for familial PHEO, 86% did not require steroid supplementation postoperatively, and the recurrence rate was only 5% at a mean follow-up of 20 months [38]. In another study by Kawasaki et al. [43], in patients that underwent BA with at least one-sided PA, 81.8% of patients successfully recovered residual adrenal function and were free from glucocorticoid supplementation after 24 months, while the remaining patients only required low corticoid doses.

The data extracted in the systematic review showed that 65.5% of patients required steroid therapy after bilateral procedures at a medium follow-up of 36 months (12–132) [9,12,16,20,35,36]. Our center’s data are apparently not in complete accordance since all patients needed steroid implementation after bilateral procedures (one side TA and other side PA) for synchronous or metachronous PHEOs. However, it should be underlined that two factors might be related to the different results. Firstly, our reported follow-up is still too short to draw definitive conclusions about the long-term need of steroid therapy (2 of our patients have 1 and 3-months follow-ups, respectively), and 3 patients require less than 20 mg/day hydrocortisone. Secondly, the median lesion size, on the PA side, was 21.5 mm (range 17–32), and the disease was multifocal in 50% of patients, predisposing for smaller remnant adrenal tissue. Moreover, our center’s post-surgical management guidelines suggest not to discontinue steroid therapy in these patients before 6 to 12 months of follow-up. Furthermore, although no adverse events were noted, post-surgical clinical conditions and technical considerations on the amount of adrenal remnant tissue lead the way of medical orientation on steroid implementation. Such considerations may explain the prolonged need of steroid therapy in our series in contrast to the literature reports [17,19].

As aforementioned, clinical outcomes after the removal of a genetic syndrome-related PHEO should not only include post-operative adrenal insufficiency but also true recurrence and metachronous disease [11].

Indeed, recurrence is considered the main risk of PA surgery: Complete adrenal medulla resection is technically impossible, and the risk of recurrence in germinal disease is thus high. Several series reported 1–23% risk of recurrence after a mean follow-up of 6–10 years after surgery [9,19,21,38]. Considering both the potential late recurrence and the young age of these patients at the time of surgery, surveillance should be carried out on a regular basis after partial procedures [19]. Regular surveillance of patients with hereditary PHEO allows recurrence detection at an early stage, thus exposing patients to fewer potential complications [8]. In these patients, the measurement of 24-h urinary excretion of fractionated catecholamines and metanephrines every 6 months is regarded as a sensitive test [19].

Data regarding the differences, in terms of recurrence rate, after open adrenalectomy vs. minimally-invasive adrenalectomy are still lacking in literature [22]. On the other hand, the real risk of recurrence has been used as an argument against anything less than TA [8]. De Graff et al. [15] reported a 45% recurrence rate after PA, which is much higher than the 3% recurrence rate reported after total BA. However, most large series showed a remnant recurrence rate of 3% to 27% [8]. Several authors even stated that PA does not carry a higher recurrence risk when compared to TA, as the risk of true local recurrence is not increased if no capsular rupture or spillage occurred during surgery [19,38]. Data from our systematic review are concordant with this evidence, as only 4 among a total of 83 patients (4.8%) developed ipsilateral recurrence of the disease. On the other hand, metachronous PHEOs occurred in 26.5% of patients, requiring further surgical treatment. Comparing such results with our personal experience, we reported only one ipsilateral recurrence after partial procedures, while three patients developed metachronous disease at a mean follow-up of 27.6 ± 29.2 months. Extra-adrenal disease was not reported in any patient.

Several limitations apply in this review for two main reasons: the first one being the retrospective nature of all but one of the studies included, with the well-known accompanying biases. The second one stems from the scarcity of available data. To date, comparative data regarding the quality of life after total and partial procedures are still lacking. Indeed, the rarity of the disease and the fragmentary nature of data in the different case series do not allow definitive comparisons between the two procedures. Further studies, evaluating not only the postoperative need of steroid supplementation but also the different impact of the two approaches on time to achieve steroid independency and quality of life, are still necessary. Furthermore, administration and dosage of post-surgical steroid therapy is not clearly specified in all the included studies. This could be explained by the lack of precise protocols on steroid therapy following TA or PA to avoid hypocortisolism’s adverse events, while current guidelines do not include precise indications for bilateral synchronous PHEOs’ optimal treatment. As a consequence, a further limitation of this review is related to the heterogeneity of followed clinical practices, patient selection, and reporting of data among centers.

Thus far, tailoring the surgical approach to the individual patient and lesion rests upon surgeons’ preferences and personal views, possibly denying a significant portion of patients less radical alternatives. The clinical implications of this study on every day practice are essential, since patients presenting with PHEO, especially in younger ages, may benefit from genetic testing at least for the major susceptibility genes. If MEN2 presence is verified, even if the lesion is unilateral at presentation, the patient should be a candidate for PA, considering the increased likelihood of presenting a metachronous contralateral lesion. Although approximately 65% of patients remain corticoid-dependent following bilateral procedures, with at least one adrenal being partial resected, the required dose of corticosteroids observed was low. Regardless of not being corticoid-independent, in our experience, reaching a dosage equilibrium and avoiding adverse events related to corticoid dosage is uncommon when PA is implemented, even unilaterally. This might positively affect quality of life long-term, although further investigation is required on this subject. Delving deeper, as PA effectiveness strongly correlates with smaller lesion size and position, patient selection should also be based on lesion characteristics.

Moving forward, the further advancement, implementation, and standardization of fluorescent techniques might enable surgeons to accurately determine PA effectiveness intraoperatively. Concurrently, a wider diffusion of robotic-assisted approaches may facilitate optimal remnant tissue volume and perfusion by minimizing tissue manipulation and enhancing surgical precision.

## 5. Conclusions

PA is a safe and valuable option in patients with hereditary PHEOs, including MEN2 patients, balancing the disease recurrence risk with steroid independency [16,21].

This specific patients’ cohort should be evaluated and treated by multidisciplinary teams, at centers with appropriate expertise, to achieve a tailored approach for each patient [22]. Perioperative management is highly specific and is best performed by a specialized surgical team well-versed in endocrine surgery and the techniques of minimally-invasive surgery [38]. In case of unilateral MEN2-PHEO, partial procedure should be considered due to the higher risk of metachronous presentation of the disease compared to ipsilateral recurrence. On the other hand, for bilateral synchronous disease, the surgical treatment (bilateral partial procedure vs. unilateral TA and contralateral PA) should be based on the patient’s and lesion’s features.

However, further studies with larger sample sizes and long-term follow-up are necessary in order to draw definitive conclusions.

## Figures and Tables

**Table 1:** Personal Experience of Partial Adrenalectomies for the Treatment of MEN2-PHEOs.

Age	Gender	Year	MEN2 Syndrome	Laterality	Procedure	Operative Time (Minutes)	POS (Days)	Follow-Up
28	Female	2017	2B (no further informations available)	Right Metachronous (Left TLA TA, 2010)	Right PA (TLA)	70	7. No post-operative complications	60 months: Right recurrence that required right CA. Hydrocortison 30 mg/day
36	Female	2020	2A, C634R	Bilateral Synchronous	Left TA + Right PA (PRA)	118	4. No post-operative complications	23 months. No recurrence. Hydrocortison 10 mg/day
36	Male	2021	2A, C634R	Left Metachronous (Right TLA TA, 2014)	Left PA (TLA)	53	3. No post-operative complications	20 months. No recurrence. Hydrocortison 20 mg/day
26	Male	2021	2A, C634R	Right	Right PA (TLA)	77	2. No post-operative complications	11 months. No recurrence. No SI
51	Female	2022	2A, C618R	Left Metachronous (Right TLA TA, 2011)	Left PA (TLA)	67	3. No post-operative complications	3 months. No recurrence. Hydrocortison 20 mg/day
39	Female	2022	2A, C634R	Bilateral Synchronous	Right TA + Left PA (PRA)	75	5. No post-operative complications	1 month. No recurrence. Hydrocortison 30 mg/day

TLA: Transabdominal Lateral Adrenalectomy. PRA: Posterior Retroperitoneoscopic Adrenalectomy. POS. Postoperative Hospital Stay. CA: Completion of Adrenalectomy.

**Table 2:** Results of Systematic Review Compared to Our Personal Experience: Partial Adrenalectomy in MEN2-pheochromocytoma patients.

Article	Study Type	PHEO	MEN2 (%)	Median Age	Laterality		First Surgery				R (Months after the Primary Tumor)	S-PHEO	M-PHEO (Months after the Primary Tumor)	Redo Surgery				FU
							TA		PA					TA		PA		
					UL	BL	UL	BL	UL	BL				IL	CL	IL	CL	
Gupta et al., 2014 [17]	Retrosp. Study	10	1 (10)	30.6	1	0	0	0	1	0	0	0	0	0	0	0	0	12
Cavallaro et al., 2011 [20]	Retrosp. Study	3	3 (100)	37	0	3	3	0	3	0	0	3	0	0	0	0	0	12
Edstrom et al., 1999 [16]	Retrosp. Study	5	5 (100)	38	3	2	4	0	3	0	0	2	3 (108)	0	0	0	3	132
Scholten et al., 2011 [12]	Retrosp. Study	61	61 (100)	33	37	24	30	22	7	2	3 (4.9)	24	17 (6.3)	3	13	0	4	16.2
Nambirajan et al., 2005 [35]	Retrosp. Study	5	1 (20)	24	1	0	0	0	1	0	1 (NR)	0	0	1	0	0	0	NR
Walz et al., 2006 [36]	Prosp. Study	134	12 (9)	41.7	4	6	5	0	7	3	0	6	2 (120)	0	0	0	2	36
Our experience		194	6 (3)	36	4	2	5	0	3	0	1 (48)	2	3 (84)	1	0	0	3	15.5

UL: Unilateral, BL: Bilateral, R: Recurrence, S: Synchronous, M: Metachronous, IL: Ipsilateral, CL: Contralateral, SI: Steroid Implementation, FU: Follow-up, NR: Not Reported.

**Table 3:** Results of Systematic Review Compared to Our Personal Experience: Clinical Outcome after Bilateral Adrenalectomy in MEN2-Pheochromocytoma Patients.

Article	Bilateral Adrenalectomies	First Surgery	Synchronous PHEO	Recurrences	Metachronous PHEO	Second Surgery	Third Surgery	SI
Cavallaro et al., 2011 [20]	3	**3 UL TA+ CL PA**	3	0	0	/		1 (33.3%)
Edstrom et al., 1999 [16]	5	**2 UL TA + CL PA**	2	0	0	/		1 (20%)
		2 UL TA	0	0	2	**2 CL PA**		
		1 UL PA	0	0	1	**1 CL TA**		
Scholten et al., 2011 [12]	42	**22 BL TA**	22	0	0	/		35 (83%)
		**2 BL PA**	2	2	0	**2 IL TA**		
		13 UL TA	0	0	13	**11 CL TA** **2 CL PA**		
		5 UL PA	0	0	4	**2 CL TA** **2 CL PA**	/	
			0	1	0	**1 BL TA**	/	
Walz, 2006 [36]	8	**3 BL PA**	3	0	0	/	/	1 (25%)
		**3 UL TA + CL PA**	3	0	0	/	/	
		2 UL TA	0	0	2	**2 CL PA**	/	
Our experience	5	**2 UL TA + CL PA**	2	0	0	/	/	5 (100%)
		3 UL TA	0	1	3	**3 CL PA**	**1 IL TA**	

UL: Unilateral, BL: Bilateral, R: Recurrence, S: Synchronous, M: Metachronous, IL: Ipsilateral, CL: Contralateral, SI: Steroid Implementation.

## Data Availability

The data presented in this study are available on request from the corresponding author. The data are not publicly available due to privacy and ethical restrictions.
